# Assessment of Trading Partners for China's Rare Earth Exports Using a Decision Analytic Approach

**DOI:** 10.1371/journal.pone.0102870

**Published:** 2014-07-22

**Authors:** Chunyan He, Yalin Lei, Jianping Ge

**Affiliations:** 1 School of Humanities and Economic Management, China University of Geosciences, Beijing, China; 2 Key Laboratory of Carrying Capacity Assessment for Resource and Environment, Ministry of Land & Resources, Beijing, China; Cinvestav-Merida, Mexico

## Abstract

Chinese rare earth export policies currently result in accelerating its depletion. Thus adopting an optimal export trade selection strategy is crucial to determining and ultimately identifying the ideal trading partners. This paper introduces a multi-attribute decision-making methodology which is then used to select the optimal trading partner. In the method, an evaluation criteria system is established to assess the seven top trading partners based on three dimensions: political relationships, economic benefits and industrial security. Specifically, a simple additive weighing model derived from an additive utility function is utilized to calculate, rank and select alternatives. Results show that Japan would be the optimal trading partner for Chinese rare earths. The criteria evaluation method of trading partners for China's rare earth exports provides the Chinese government with a tool to enhance rare earth industrial policies.

## Introduction

### Current states of RE supply and demand in China and the world

Rare earths (REs), despite the name, are relatively abundant in the earth's crust. According to the US Geological Survey (2012), the global rare earth oxide (REO) reserves were over 110 million tons in 2010 [1]. Recently, with the improvement of RE ore mining and processing technologies, and development of new energy fields, output has soared. Between 2001 and 2010, the global RE output increased 51% [2]. Concomitantly, there is also a growing RE world market demand. Besides China, the world's largest RE consumer, the world's top 4 consumers are Japan, USA, France, and Korea, respectively [3]. Moreover, the European countries of the Netherlands, Germany and UK also consume large amounts of RE annually. Top uses for RE on the world market include catalysts, magnets and metal alloys. Such uses are characterized by high specificity and high unit value. Thus, demand for REs will continue to increase with the development of the global economy and high-tech applications.

**Figure 1:**
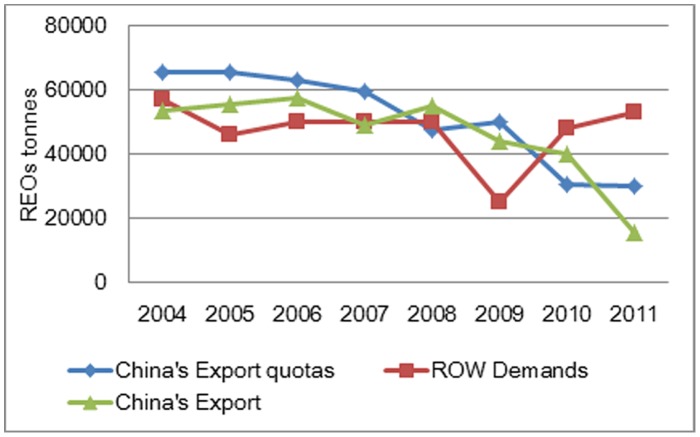
China's RE Supply and Demand in the World between 2004 and 2010.

Given its abundant RE production, consumption and trade impact, China is key player in world market. **Table 1** shows that China holds 48% of the world RE reserves and produces 97% of the world's supplies, 64% of which is for the domestic consumption and 12% of which is for legal export. In other words, China is supplying almost all of the world RE demands, but holds only 48% of the world RE reserves as the RE market continues to grow. Overproduction will accelerate the exhaustion of RE resources, and may well create a black market in RE products. According to the Information Office of the State Council, P.R.C., the 2011 volume of RE products imported from China were 1.2-fold greater than the volume exported [4]. Thus, world RE trade is comprised of mainly Chinese exports to the rest of world (ROW). However, China's advantage in RE resources does not equally apply to domestic industrial applications. Beginning in 1999, China imposed RE export quotas. The policy has had limited success [5]. Since 2010, China's RE exports have exceeded export quotas, which is showed in **Figure 1** [6], [8]–[14] (data of ROW demands are estimated). Furthermore, if smuggling volumes are added to China's RE export totals, the total amount of RE leaving China will significantly exceed export quotas. Therefore China's RE trade cannot continue to satisfy the normal international market demand.

**Table 1:** RE Reserves, Production, Consumption and Export in China and the World in 2011.

Country/region	Reserves^a^	% of total	Mine Production^ae^	% of total	Consumption^b^	% of total	Export^b^	% of total
China	55,000	48	130	97	83,110	64	15,700	12
World	113,778	-	133.58	-	158,200	-	-	-

a Data in thousand metric tons of REO content; ^b^ Data in tons of REO content; ^e^ Estimated.

Source: [1], [6], [7].

### A review of RE international trade study

The recent RE literature has focused on a growing RE supply crisis and soaring Chinese RE export prices. Three research threads have emerged: (1) A critical assessment of RE supply risk. (2) RE international pricing. (3) China's RE trade policies. Most papers in Thread (1) have dealt with global RE market supply and an assessment of China's RE short supply risk. Some authors have used a matrix approach linking individual minerals with “supply disruption” and “supply risk” dimensions [15], [16]. Researchers considering Thread (2) have used economic models and quantitative methods to understand China's RE pricing power in world markets [17], HHI [18] and BPI [19]. Finally, Thread (3) research has examined the role of trade policy in the global RE market. Much of this work has focused on export quotas within a WTO frame work [20]–[23].

The most recent literature focuses only on current conditions and or qualitatively discusses the effect of China's RE trade policies on global RE supply-demand balances. The lack of RE data collection is also a significant limiting factor. Furthermore, systematic evaluative criteria for the selection of Chinese trading partners of RE exports are scarce indeed.

### Study purpose and framework

Simply put, Chinese RE output is insufficient to meet future world demand. China must adopt a selective RE export strategy for the rest of the world (ROW) to ensure sustainable development of China's RE resources. We suggest an optimal trading partner methodology for China's RE export trade. A Multi-attribute Decision Making (MADM) method is utilized to solve this decision-making (DM) problem. This methodology will provide crucial support data for the development of China's RE policy. Therefore, the aim of this paper is to introduce a MADM method for selecting an optimal trading partner of China's RE exports. Our preliminary results can become support for the Chinese government to enhance and improve its RE policies.

This paper is organized as follows. The next section *Materials and Methods* use our MADM methodology to develop and present a simple additive weighing (SAW) model. An evaluative criteria system is also presented which can be used to assess China's top trading partners. Section 3 *SAW Model Application in the Optimal Trading Partner Selection* presents and applies our criteria system and the SAW model for identifying and evaluating the optimal trading partner for Chinese REs. Section 4 *Discussion* discusses our study results and selection. Section 5 *Conclusions* presents what have been drawn in this study.

## Materials and Methods

### Ethic Statement

Each participant offered informed consent as to the purpose of the questionnaire and their involvement in the study. The survey was anonymous, and data from questionnaires was treated as confidential. The questionnaire did not contain any identifying information regarding participants. In China, there are not specialized IRB reviews and approval. However, we consulted the head of the Department of Science and Technology of China University of Geosciences (Beijing) and obtained the approval for this study and questionnaire and method.

### MADM method

Multi-attribute decision-making (MADM) (sometimes called Multi-criteria Decision Analysis), a rule-based method of classification for priority setting, is both a set of techniques and an approach for ranking alternatives [24]–[26]. This method has been widely used in ranking or selecting one or more alternatives from a finite number of alternatives with respect to multiple, usually conflicting, criteria or attributes [27], [28]. Hwang and Yoon classify them as (i) Multiple Attribute Decision Making (MADM), with discrete, usually limited, number of pre-specified alternatives, requiring inter and intra-attribute comparisons, involving implicit or explicit tradeoffs; and (ii) Multiple Objective Decision Making (MODM), with decision variable values to be determined in a continuous or integer domain, of infinite or large number of choices, to best satisfy the DM constraints, preferences or priorities [29]. MADM is an approach employed to solve problems involving selection from among a finite number of alternatives [30]. This paper applies a MADM method (i.e. selection), to assess and determine China's optimal RE trading partners.

A MADM method specifies how attribute information is to be processed in order to arrive at a choice. Further, the method requires both inter- and intra-attribute comparisons, and explicit decision tradeoffs [30]. Each MADM decision table (also called decision matrix) consists of four main elements: (a) alternatives, (b) attributes, (c) weight or relative importance of each attribute, and (d) measures of performance for alternatives with respect to the attributes [30]. Given the decision table information and a decision-making method, the task of the decision maker is to find the best alternative and/or to rank the entire set of alternatives [30].

Alternatives within a MADM framework can be evaluated. Alternatives are then ranked in accordance to the importance of each attribute for a decision maker [31]. The following three steps are used in any DM method involving numerical analysis of alternatives [32]:

1. determining the relevant attributes and alternatives (i.e. establishing an evaluation criteria system);
2. attaching numerical measures to the relative importance of the attributes and to the impacts of the alternatives on these attributes (i.e. determining attribute weights);
3. processing the numerical values to determine a ranking of each alternative (i.e. calculating and ranking with DM methods)

Only the last step actually involves a DM method.

### Establishment of the evaluation hierarchy

#### A review of mineral resources international trade assessment criteria

A summary of evaluation criteria for mineral resources' international trade is illustrated in **Table 2**. It is obvious that politics, economics and industrial structure are major dimensions of international trade in mineral resources. Resource dimension becomes important only when resource endowments such as reserves and output are crucial. The market dimension criteria largely pertain to the existing economics or industry dimensions though isolating them may have value in certain international trade situations. With the exception of political issues, the criteria can be quantified with limited difficulty. Qualitative methods such as the Fuzzy Evaluation and Delphi Methods could be used to offer insights into political dimensions.

**Table 2:** Summary of Evaluation Criteria for Mineral Resources International Trade.

Dimensions	Criteria	Literatures
Resources	Reserves	[33], [34]
	Output	[34]
	Resources environment risk	[33], [35], [36]
Politics	Stratigic stockpile	[33]
	Security of transport transport routes	[33], [37]–[39]
	Relationship with China	[37]–[39]
	Political environment	[37]–[39]
	Foreign capital policities	[16], [35], [36]
	Fiscal policies	[38], [40]
	Regime	[38], [40]
	Political risk	[16], [38], [39], [41]
Economics	Economic risk	[35], [41]
	GDP	[37], [38]
	Ratio of dependence on foreign trade	[37], [42]
	profit and taxes	[42]–[44]
	economic freedom	[38]
	economic competitativeness	[38]
	Economic systems	[40]
	Total import-export value	[45]
	Contribution of exports to GDP growth	[45]
	Economic benefit	[44]
Markets	Import/market potential	[46], [47]
	Import security	[46]
	International market share	[48]
	Concentration Ratio (CRn)	[34], [42]
	Market capacity	[47]
	International market price	[33], [39]
Industry	Industrialinternational competitativeness	[42], [48]
	Structure of import and export goods	[45]
	Trade structure	[44], [46]
	Industrial structure	[44], [46]
	Trade size	[44], [46]
	Basic Availability	[16]
	Competing Technology Demand	[16]
	Codependence on Other Markets	[16]
	Industry protection	[39]
	Size of industry	[43]

Although the evaluation dimensions and main criteria set forth in **Table 2** are not perfect, they can be used to develop the optimal trading partner selection framework.

In our study, an evaluation criteria system (i.e. hierarchical structure) was established to determine the optimal trading partner for China's RE export based on MADM procedure. Three dimensions comprising a total of 10 criteria, 24 sub-criteria are considered and 7 alternatives are evaluated.

#### Construction of the hierarchical structure

The two-dimensional criteria outlined in **Table 2** do not directly take into account the current reality of RE supply and demand in China and the ROW. Particularly absent were dimensions dealing with national security issues important in China today. To address this, we interviewed a panel of experts from China University of Geosciences-Beijing (CUGB) and Chinese Academy of Land & Resource Economics (CALRE) who helped us refine and cluster the criteria from **Table 2** into a more comprehensive matrix. Experts came from the following fields: industrial economics, mineral resources management and policy research, mineral resources research.

**Figure 2:**
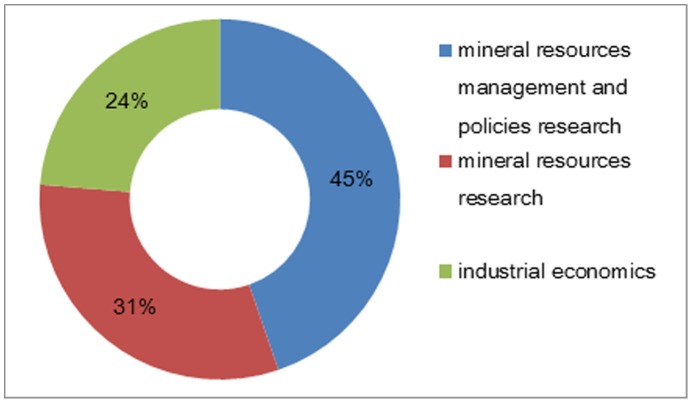
Evaluation Hierarchy for Trading Partner Assessment of China's RE Export Trade.

Criteria are tools for assessing alternatives, reflecting all the concerns and relevant issues to the decision problem [49]. We establish a 3 dimensional, 10 criteria, 24 sub-criteria and 7 alternatives hierarchical evaluative structure shown in **Figure 2** **.** It is suggested by the literature summed in **Table 2** together with the Delphi approach which is designed to elicit the views of a panel of experts [50]. The essence of the technique is fairly straightforward [50]. The Delphi panel suggested we introduce alternatives to our criteria. The alternatives represent the world's seven top RE consumption countries.

Using this hierarchical structure, the “optimal trading partner” for China's RE export trade can be defined by the following three characteristics:

- political relationships: a country exhibits a stable political situation, safe RE transport routes, and good political relationships with China;
- economic benefits: a country has positive economic environment for international trade, significant RE demands, stable RE import prices, and import policies that encourage RE markets;
- industrial security: a country enjoys significant real growth in domestic output, and its RE demand matches China's supply of RE.

### Method of determining attribute weights

#### Direct-radio method

The methods of determining attribute weights fall into two categories: objective weighing methods such as Entropy, and subjective weighing methods such as Direct-radio and AHP. Objective weighing methods are commonly used weighing schemes for those attributes that are easily quantified. Subjective weighing methods more commonly applied to those attributes that are more difficult to quantify. Since many of our attributes fall into the latter category of difficult to quantify, we use the direct-radio method (one of the subjective weighing methods). Thus our expert panel directly determined the weights of each attribute. According to this method, the corresponding eigenvector is:
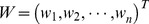
(1)

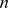
 is the number of attributes.

In order to compare, the values of the weights should be normalized:
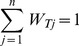
(2)where 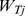 is the 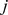-th attribute's aggregate weights.

Experts directly give the value of each attribute of the sub-criteria level according to its importance using a 9-point scale. 1 is the least important while 9 is the most. Means are then calculated as:
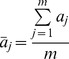
(3)where 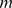 is the number of experts; 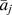 is the mean of the 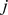-th attribute from 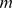 expert assessment values.

Then the criteria means of the other levels are also worked out based on their sub-level's mean values by this average method. Take the mean calculation of C_11_ and D_1_ as the example.
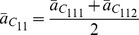
where 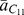 is the mean of criterion C_11_ and both 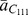 and 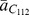 are the means of criterion C_11_'s sub-level criteria (namely C_111_ and C_112_).

In the same way, the mean of criterion D_1_ can be calculated, namely 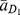 as follows.
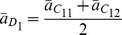



After obtaining the means as outlined above, all mean values must be normalized as below:

1. normalization procedures

The normalization procedure requires that the total value for all its attributes associated with a certain criterion equal one:
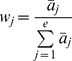
(4)where 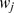 is the 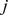-th attribute's normalized weights; 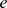 is the attribute numbers the 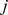-th attribute's upper level criterion contains.

(2) aggregate weights

The aggregate weight is obtained by multiplying the appropriate dimension weight times the appropriate criteria weight times the appropriate sub-criteria weight, and set it as 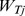. The Equation is:
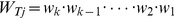
(5)where 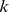 is the number of level. In general, the first level, as the top goal level, always has only one attribute, so 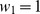. Here, take the aggregate weights calculation of C_111_ as the example.
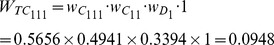



#### Estimating weights of dimensions and criteria

1. Questionnaire design

The questionnaire includes 24 items (i.e. 24 sub-criteria of the hierarchy structure in **Figure 2**) and our experts assign values according to a 9-point scale importance scale.

(2) Experts choice

Experts came from the following fields: industrial economics, mineral resources management and policy research, mineral resources research.

(3) Issuance and callback of questionnaires

**Figure 3:**
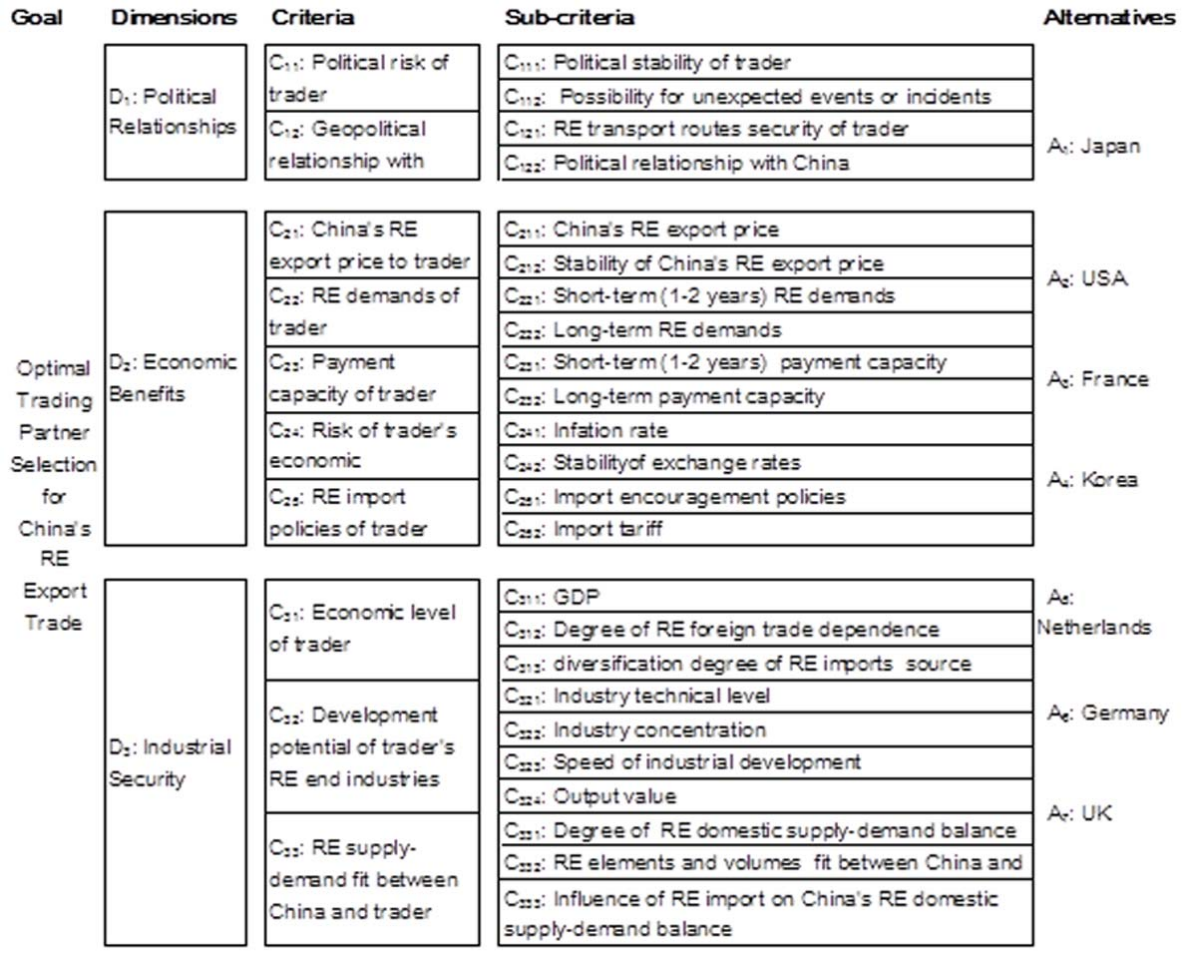
Distribution Ratios of Experts from Different Research Fields.

Eighty questionnaires were issued either by email or regular mail. Seventy-six useable surveys were returned. Thus the effective return ratio was 95%. Of the effective 76 questionnaires, 34 were from experts in the mineral resources management and policies research field, 24 were from experts in the mineral resources research field, and 18 were from experts in the industrial economics field (**Figure 3**). The highest return ration was from the mineral resources management and policies research field. This field includes both management and policy studies. In practice it is difficult to distinguish these two areas. Thus the higher representation from this expert field is reasonable.

(4) Normalizing 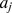 values and calculating the synthesis weights 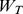

The evaluation results are normalized using Equation 4. Then the aggregate weights of each level are calculated with Equation 5. **Table 3** lists the weights of each level.

**Table 3:** Aggregate Weights of Each Level.

Weights for dimensions	Weights for criteria	Weights for sub-criteria	Aggregate weights
*w_2_*	*w_3_*	*w_4_*	*W_T_*
D_1_: 0.3394	C_11_: 0.4941	C_111_: 0.5656	0.0948
		C_112_: 0.4344	0.0728
	C_12_: 0.5059	C_121_: 0.4615	0.0792
		C_122_: 0.5385	0.0925
D_2_: 0.3463	C_21_: 0.2175	C_211_: 0.5146	0.0388
		C_212_: 0.4854	0.0366
	C_22_: 0.2128	C_221_: 0.4791	0.0353
		C_222_: 0.5209	0.0384
	C_23_: 0.2013	C_231_: 0.4800	0.0335
		C_232_: 0.5200	0.0362
	C_24_: 0.1713	C_241_: 0.4715	0.0280
		C_242_: 0.5385	0.0319
	C_25_: 0.1971	C_251_: 0.4968	0.0339
		C_252_: 0.5032	0.0343
D_3_: 0.3143	C_31_: 0.3180	C_311_: 0.2417	0.0242
		C_312_: 0.4068	0.0407
		C_313_: 0.3515	0.0351
	C_32_: 0.3251	C_321_: 0.2579	0.0264
		C_322_: 0.2388	0.0244
		C_323_: 0.2573	0.0263
		C_324_: 0.2460	0.0251
	C_33_: 0.3569	C_331_: 0.2988	0.0335
		C_332_: 0.3459	0.0388
		C_333_: 0.3553	0.0399

In this hierarchy structure, there are four levels, so 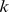 in Equation 5 is 4. Thus, 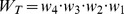. Here, the goal is only one, so 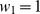.

### Method of calculating each alternative: simple additive weighing (SAW) model

The simplest and most widely used MADM method is a SAW model that calculates weighted scores for each option [29], [30], [51]. Like other multi-attribute models, the SAW model is used to evaluate, rank and select the most appropriate choice among alternatives [52]. A SAW model is a linear evaluation model and is constructed using an additive utility function (AUF).

A MADM problem is defined as a set of actions (alternatives) called 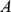 for which there is a consistent family 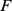
of 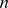 attributes 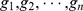
_._ One wishes to assess and rank the actions of 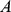 from best to worst and determine a subset of actions considered to be the best with respect to the 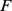 attributes [53], [54]. Historically, assessing a model of overall individual preferences) results in the aggregation of all criteria into a unique utility function [55]–[57] like (6) below.
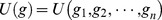
(6)


The utility function is additive when it is of the form
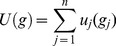
(7)where 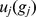 is the 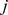-th attribute's marginal utility.

A weighted sum form of the utility function can be achieved by simply including the appropriate weights as shown in (8) below:
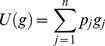
(8)where each attribute's marginal utility is totally determined by the attribute 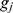 and weight 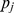.

A SAW version of model (8) for Chinese RE trading partners could take the form:
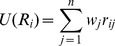
(9)where 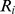 is one alternative (trading partner) and 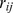 is the value of the 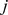-th attribute for the 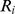
alternative, and 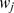 is the weight of the 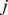-th attribute.

Equation 9 requires attribute values which must be both numerical and comparable. That is to say, the decision attributes must be expressed in identical units of measure (e.g., only dollars, only pounds, only seconds, etc.) [30]. When both quantitative and qualitative attributes are used in a SAW model, normalization is required to convert all values into non-dimensional quantities 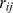 (i.e. no unit) thus making them comparable.

Before non-dimensionalization, every attribute should be uni-directional. In this paper, three types of attributes are used: beneficial attributes, non-beneficial attributes and target value attributes. Higher values for beneficial attributes (e.g., profit) are more desirable than lower values for the given decision-making problem [30]. By contrast, non-beneficial attributes (e.g., cost) suggest lower values are more desirable [30]. For target value attributes the closer the attribute value is to some target value the more preferable. Thus all the attributes are converted into the beneficial attributes.

Non-beneficial values are converted into beneficial ones using the following transformation [58]:
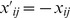
(10)and the target values are converted into beneficial ones by the following transformation [58]:
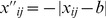
(11)where 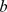 is the target values of the 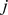
–th attribute, thus a constant.

Ye pointed out that a mean normalization method is the best choice when attribute values are all objective [58]. This method maintains the degree of variation from the original variable while eliminating the effects of dimensions and order of magnitude. Further, the converted values are not dependent on the standard deviation of original variables, but their coefficient of variation [59]. The mean normalization method uses the following transformations:
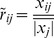
(12a)

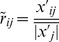
(12b)

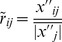
(12c)where 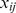 is the original values of the 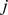-th attribute for the 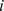-th alternative and 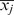 is its mean; 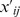 and 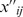 are respective the processed values in Equations 10 and Equations 11 and 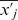 and 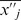 are their means; 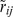 is the normalized value of the 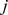-th attribute for the 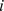-th alternative. Each of the three transformations is applied as follows: beneficial attributes use equation 12a; non-beneficial use equations 12b; and equation 12c applies to target value attributes.

Using the above two steps, the 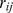 has been converted into non-dimensional values 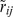. Of course, the weight 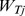 should be normalized using equation 5. In that case, the SAW model will take the following form:
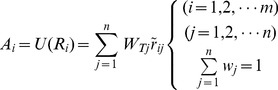
(13)where 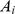 is the overall or composite values for the 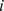-th alternative; 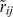 represents the normalized value of 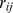; 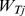 is the 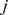-th attribute's aggregate weights.

The alternative with the highest value of 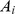 is considered as the best alternative.

### SAW Model Application in the Optimal Trading Partner Selection

Using the MADM method, an assessment of seven top trader partners of China RE exports between 2006 and 2010 is conducted according to the criteria evaluation system established and showed in **Figure 2**.

In our hierarchical structure, all the criteria of dimension 2 and 3 can be quantified. Our data includes five-year values criteria for all alternatives (seven countries) from 2006 to 2010. These original data are from Statistical Yearbook of China (http://www.stats.gov.cn/tjsj/ndsj/), CCS (http://www.chinacustomsstat.com/), USGS (http://www.usgs.gov/) and CRE (http://www.cre.net/). The criteria values of dimension 1 cannot be quantified so a Fuzzy MADM method is used. This method systematically converts linguistic terms into their corresponding fuzzy numbers [30]. In this paper, a 5-point scale having the linguistic terms low, below average, average, above average, and high used [30]. Using computer processing, the values of dimension 1 are obtained. Meanwhile, this method is also utilized for a sub-criterion called import encouragement policies, but the only difference is that a 2-point scale (0 and 1) is used to have the linguistic terms no (0) and yes (1).

After a series of nondimensionalizations to this original statistical data with Equation 10, 11 and 12, the nondimensionalized value 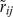 is listed in **Table 4**.

**Table 4:** Nondimensionalized Values of Each Alternative.

Sub-criteria	A_1_: Japan	A_2_: USA	A_3_: France	A_4_: Korea	A_5_: Netherlands	A_6_: Germany	A_7_: UK
C_111_	1.0000	1.2500	1.0000	0.7500	1.0000	1.0000	1.0000
C_112_	1.2174	0.3043	0.9130	0.9130	1.5217	1.2174	0.9130
C_121_	1.1200	1.4000	0.8400	1.1200	0.8400	1.4000	0.2800
C_122_	0.6774	1.1290	1.1290	0.6774	1.1290	1.1290	1.1290
C_211_	1.9798	0.8662	0.6717	1.3434	0.7071	0.6187	0.8131
C_212_	−1.9798	−0.8662	−0.6717	−1.3434	−0.7071	−0.6187	−0.8131
C_221_	2.7962	1.7148	0.5235	0.3982	0.0578	1.4199	0.0897
C_222_	2.6464	1.5871	0.8118	0.4860	0.2449	1.1055	0.1183
C_231_	1.5065	0.5782	0.8964	2.9029	0.2661	0.6759	0.1740
C_232_	1.0008	0.9983	1.0000	1.0012	0.9990	0.9997	1.0010
C_241_	1.5169	−4.1189	−0.0925	−3.0874	−1.3578	−0.3734	0.5130
C_242_	−0.5967	0.0000	0.0000	−6.4033	0.0000	0.0000	0.0000
C_251_	0.0000	0.0000	1.4000	1.4000	1.4000	1.4000	1.4000
C_252_	0.0000	−1.4300	−1.0350	−1.4300	−1.0350	−1.0350	−1.0350
C_311_	1.2182	3.4271	0.6369	0.2001	0.1904	0.8045	0.5227
C_312_	0.4048	0.4191	0.7749	1.4458	2.0966	1.0367	0.8221
C_313_	−1.0348	−1.2174	−1.0957	−0.6696	−0.6696	−1.2783	−1.0348
C_321_	1.0874	1.5195	0.0000	0.9193	1.3293	1.1668	0.9778
C_322_	1.1888	0.7102	0.9831	1.1184	0.7680	1.3997	0.8318
C_323_	−0.1293	0.2596	−0.1630	2.6533	−0.0636	4.4654	−0.0223
C_324_	3.4390	1.7858	0.4401	0.1541	0.1207	0.7683	0.2919
C_331_	−2.6983	−1.6467	−0.1102	−0.5332	−0.0340	−1.9489	−0.0288
C_332_	−0.2925	−0.2190	−1.2748	−0.9272	−0.7067	−2.6751	−0.9047
C_333_	2.7962	1.7148	0.5235	0.3982	0.0578	1.4199	0.0897

At last, compositing the synthetic weights in **Table 3** and the normalized values in **Table 4** with Equation 13, the final score 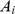 of each alternative is shown in **Table 5**. Take the score of Japan for criterion C_111_ as the example.
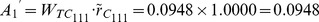



**Table 5:** Composite Value of Each Alternative with SAW Model.

Sub-criteria	A_1_: Japan	A_2_: USA	A_3_: France	A_4_: Korea	A_5_: Netherlands	A_6_: Germany	A_7_: UK
C_111_	0.0948	0.1186	0.0948	0.0711	0.0948	0.0948	0.0948
C_112_	0.0887	0.0222	0.0665	0.0665	0.1109	0.0887	0.0665
C_121_	0.0887	0.1109	0.0666	0.0887	0.0666	0.1109	0.0222
C_122_	0.0626	0.1044	0.1044	0.0626	0.1044	0.1044	0.1044
C_211_	0.0767	0.0336	0.0260	0.0521	0.0274	0.0240	0.0315
C_212_	−0.0724	−0.0317	−0.0246	−0.0491	−0.0259	−0.0226	−0.0297
C_221_	0.0987	0.0605	0.0185	0.0141	0.0020	0.0501	0.0032
C_222_	0.1016	0.0609	0.0312	0.0187	0.0094	0.0424	0.0045
C_231_	0.0504	0.0193	0.0300	0.0971	0.0089	0.0226	0.0058
C_232_	0.0363	0.0362	0.0362	0.0363	0.0362	0.0362	0.0363
C_241_	0.0424	−0.1152	−0.0026	−0.0864	−0.0380	−0.0104	0.0143
C_242_	−0.0191	0.0000	0.0000	−0.2045	0.0000	0.0000	0.0000
C_251_	0.0000	0.0000	0.0475	0.0475	0.0475	0.0475	0.0475
C_252_	0.0000	−0.0491	−0.0355	−0.0491	−0.0355	−0.0355	−0.0355
C_311_	0.0294	0.0828	0.0154	0.0048	0.0046	0.0194	0.0126
C_312_	0.0165	0.0170	0.0315	0.0588	0.0852	0.0422	0.0334
C_313_	−0.0364	−0.0428	−0.0385	−0.0235	−0.0235	−0.0449	−0.0364
C_321_	0.0287	0.0400	0.0000	0.0242	0.0350	0.0307	0.0258
C_322_	0.0290	0.0173	0.0240	0.0273	0.0187	0.0342	0.0203
C_323_	−0.0034	0.0068	−0.0043	0.0698	−0.0017	0.1174	−0.0006
C_324_	0.0864	0.0449	0.0111	0.0039	0.0030	0.0193	0.0073
C_331_	−0.0904	−0.0552	−0.0037	−0.0179	−0.0011	−0.0653	−0.0010
C_332_	−0.0113	−0.0085	−0.0495	−0.0360	−0.0274	−0.1038	−0.0351
C_333_	0.1114	0.0683	0.0209	0.0159	0.0023	0.0566	0.0036
Synthesis scores	0.8095	0.5414	0.4659	0.2929	0.5039	0.6589	0.3958

From **Table 5**, the synthetic score of each alternative (country) is as follows:
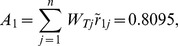


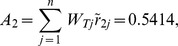


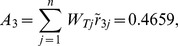


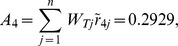


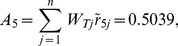


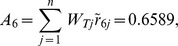


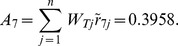



Thus, the ranking result is 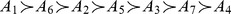 (i.e. 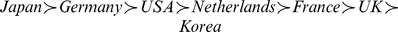). Japan is the optimal trading partner for China's RE export trade based on this assessment result.

## Discussion

The result that Japan is the optimal trading partner for China's RE exports should be understood in the following context. Japan is the overall optimal trading partner given the three assessment dimensions. Though evaluation scores of its political relationships with China are lower than the other countries, its high scores in other criteria result in a top of overall scores.

Of course, the above study methods have several limitations. The results of weights of criteria determined by means of the direct-radio method may well be influenced by subjective factors such as experts' experience and attitude. Thus, a different expert pond could well lead to different study results. However, the aim of this paper is to introduce a MADM method for selecting the optimal trading partner and presenting the initial study results which may be the basis for research, especially RE policy study.

**Figure 4:**
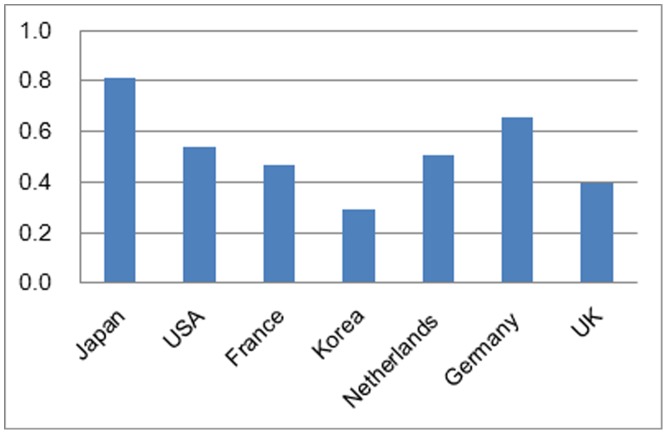
Overall Score of Each Country.

**Figure 5:**
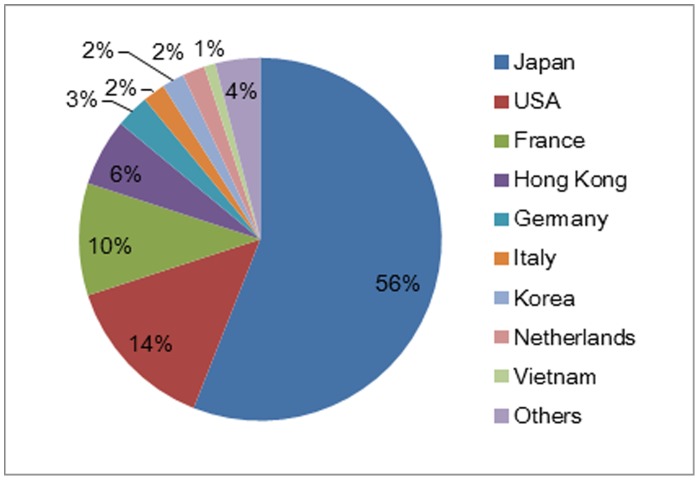
Markets of China's RE Export in 2011.

Japan is the top RE demand (consumption) country in the ROW overall ranking (**Figure 4**). This research result is consistent with the current reality of Japan as the biggest market (56%) for China's RE export in 2011 showed in **Figure 5** **** [4]. Japan has high RE import volumes and low RE import tariffs both of which directly result in a high score. However, a brief word about political criteria is important. Using Japan as an example, certain political issues seem clear. So future research could look at these issues.

Recently, unstable relationships have developed between both countries and frequent conflicts have resulted in a low score in the political relationships dimension. Nonetheless, in the long run, we do not expect these issues to affect the position of Japan as the optimal country for China's RE export trade. We make this judgement for the following reasons:

First, Japan's proximity to China results in lower shipment cost and short transportation routes.

Second, Japan has no domestic RE supplies. While Japan is actively searching for new suppliers, some heavy rare earth elements (HREE) like Dy China is the sole supplier. Furthermore, the cost of R&D alternatives is far higher than the import price of China's Res. Thus, such substitutes generally are less effective than the use of China REs.

Third, REs are a critical material for hi-tech products and Japan is a leading technology user for products such as electric and hybrid vehicle motors, electronics and rechargeable batteries. Given comparative advantage, China has the rich REs, and Japan has advanced technologies of using REs, thus both countries' trade is mutual complementary and mutual beneficial for each party. From this reason, RE trade will bring mutual economic benefits for both parties. This confirms the high score in the second dimension of economic benefits.

Finally, in the industrial security, there is a high RE industrial fit in both countries' trade. China's RE production capacity exceeds global RE demands. Therefore, exporting more REs to a high-demand country like Japan (especially the light rare earth elements (LREE) exports) can maintain a balance between RE supply and demand.

Given our research methodology, whether geographic location, economic benefits or industrial security, Japan will continue to be the optimal country for China's RE export trade.

## Conclusions

In this paper a hierarchical structure for assessing trading partners of China's RE exports is established. In the field of RE international trade assessment, it is the first time to establish such a systematic evaluation criteria system, so this unique evaluation model provides the high-value reference for the other scholars. Political relationships, economic benefits as well as industrial security of RE international trade are all assessed. Thus, the criteria are systematic and contextual. Meanwhile, the use of respected experts to assign weights of all the criteria is supportable. Therefore, the criteria system is feasible and operable. Then SAW model is used to calculate and rank the scores of this criteria evaluation system. In this model, all the data including linguistic terms, fuzzy numbers and precise numerical values are converted reasonably by FMADM method and nondimensionalization method. Using this method, an assessment of seven top trader partners of China RE exports between 2006 and 2010 is conducted and a result is gotten that Japan is the optimal trade partner. At last, we discuss the reliability and the results.

However, there is no similar hierarchy structure to which we can compare our results, so this is an exploratory study and the criteria system needs be improved in future. The criteria may be filtered by methods like Principal Component Analysis (PCA) and Factor Analysis in future studies.

Furthermore, this paper mainly focuses on the criteria system construction and the application of SAW model. In future, based on this evaluation results, an optimal configuration study of RE supplies to these seven trading partners can be further conducted.
